# Safety and accuracy of cardiac magnetic resonance imaging combined with low-dose dobutamine stress-testing in patients with congenital heart disease

**DOI:** 10.1186/1532-429X-11-S1-P27

**Published:** 2009-01-28

**Authors:** Saskia E Luijnenburg, Daniëlle Robbers-Visser, Jochem van den Berg, Jolien W Roos-Hesselink, Adriaan Moelker, Willem A Helbing

**Affiliations:** Erasmus MC, Rotterdam, Netherlands

**Keywords:** Congenital Heart Disease, Cardiac Magnetic Resonance, Dobutamine, Flow Measurement, Short Axis

## Objective

to report our experiences with low-dose dobutamine stress cardiovascular magnetic resonance (DCMR) imaging in patients with congenital heart disease (CHD) and to assess the intra-observer and interobserver variability of biventricular function, volumes, and mass.

## Background

Stress-testing is an important tool to obtain additional information on ventricular and vascular function in patients with acquired or congenital heart disease. Low-dose DCMR imaging in small groups of patients with CHD revealed ventricular and vascular dysfunction that is not apparent at rest. There are no reports in a large series of patients on the safety and accuracy of low-dose DCMR imaging.

## Methods

inclusion of all patients who underwent low-dose DCMR in our institution. Acquisition of a short axis set and flow measurements at rest, and during dobutamine administered at 7.5 μg/kg/min maximum. Intra-observer and interobserver variability (coefficient of variation) was determined for biventricular function, volumes, and mass.

## Results

In 91 patients 110 studies were performed (63 male subjects, 54 tetralogy of Fallot, 37 Fontan patients, youngest age at study 6.8 years). In 3 patients minor side effects occurred (vertigo, headache, bigeminy). In 10 patients dobutamine was lowered to 5 μg/kg/min because of an increase in heart rate of >150% baseline, although well tolerated. Intra-observer variability was between 2.3 and 8.7% for rest and stress measurements. Interobserver variability was between 3.6 and 10.5% at rest. With stress-testing, the coefficient of variation for biventricular ESV increased significantly to ≥ 15%, while it remained < 10% for the other variables.

## Conclusion

In patients with various types of CHD low-dose DCMR imaging is feasible, safe, and can be performed from 7 years of age. Intra-observer variability is low for rest and stress measurements. With stress-testing, interobserver variability of biventricular ESV increases significantly.

